# Radiotherapy Dosing for Locally Advanced Non-Small Cell Lung Carcinoma: “MTD” or “ALARA”?

**DOI:** 10.3389/fonc.2017.00205

**Published:** 2017-09-21

**Authors:** Nitin Ohri

**Affiliations:** ^1^Radiation Oncology, Albert Einstein College of Medicine, The Bronx, NY, United States; ^2^Radiation Oncology, Montefiore Medical Center, The Bronx, NY, United States

**Keywords:** locally advanced NSCLC, radiotherapy, dose–response relationship, radiation, chemoradiotherapy, lung cancer

## Abstract

Locally advanced non-small cell lung cancer (LA-NSCLC) is typically treated with thoracic radiotherapy, often in combination with cytotoxic chemotherapy. Despite tremendous advances in the evaluation, treatment techniques, and supportive care measures provided to LA-NSCLC patients, local disease progression and distant metastases frequently develop following definitive therapy. A recent landmark randomized trial demonstrated that radiotherapy dose escalation may reduce survival rates, highlighting our poor understanding of the effects of thoracic radiotherapy for LA-NSCLC. Here, we present rationale for further studies of radiotherapy dose escalation as well as arguments for exploring relatively low radiotherapy doses for LA-NSCLC.

## Background

Non-small cell lung cancer (NSCLC) is the leading cause of cancer mortality in the United States and worldwide, causing over one million deaths each year (1). Approximately one-third of NSCLC patients are diagnosed with locally advanced disease, which may be defined as stage III disease or unresectable stage II disease (2). For locally advanced non-small cell lung cancer (LA-NSCLC), the standard treatment approach is conventionally fractionated (1.8–2.0 Gy/day) radiotherapy to a dose of approximately 60–66 Gy with concurrent, platinum-based chemotherapy. This treatment approach yields median survival times of only 16–30 months. Randomized trials have tested changes or additions to systemic therapy (3–7), radiotherapy dose escalation (6), and the addition of surgical resection (8) but have failed to improve overall survival for this patient population.

In this review, we will focus on the question of radiotherapy dosing for LA-NSCLC. Dozens of trials have sought to identify the optimal dosing schedule through modifications of the total radiotherapy dose, the daily radiotherapy dose, and treatment frequency (9, 10). However, tremendous uncertainty persists regarding the optimal radiotherapy regimen for LA-NSCLC. As an infinite number of radiotherapy schedules could be an envisioned, we will simplify our discussion by considering two opposing viewpoints: “maximum tolerated dose” (MTD) and “as low as reasonably achievable” (ALARA).

Maximum tolerated dose is defined by the National Cancer Institute as follows: “The highest dose of a drug or treatment that does not cause unacceptable side effects. The MTD is determined in clinical trials by testing increasing doses on different groups of people until the highest dose with acceptable side effects is found.” (11) The United States Nuclear Regulatory Commission states that “ALARA is an acronym for *as low as (is) reasonably achievable*, which means making every reasonable effort to maintain exposures to ionizing radiation as far below the dose limits as practical, consistent with the purpose for which the licensed activity is undertaken…” (12) ALARA is most often used in the context of environmental or occupational radiation exposure. For the purposes of this exercise, we will consider ALARA to represent the delivery of the lowest possible radiotherapy dose for LA-NSCLC that does not compromise local disease control probability.

## Fact: Disease Progression Following Chemoradiotherapy for LA-NSCLC is Common

### Supporting MTD

Chemoradiotherapy for LA-NSCLC yields local control rates of only 40–66% (6, 13–17). At least 75% of LA-NSCLC patients will succumb to their disease (6). While distant disease progression is a competing risk for LA-NSCLC that may theoretically detract from the importance of local control, there is high-level evidence that improving local control will directly improve survival rates. In a meta-analysis of six randomized trials comparing concurrent chemoradiotherapy to sequential chemoradiotherapy, the use of concurrent chemoradiotherapy increased the 5-year locoregional control rate by 6% at 5 years and improved the overall survival rate by 5% at 5 years, without reducing the frequency of distant metastasis (18). Thus, there seems to be a nearly 1:1 ratio linking locoregional disease control and overall survival in LA-NSCLC. This may be compared with the 4:1 ratio that has been established in the treatment of breast cancer with postoperative radiotherapy (19). The importance of local control in LA-NSCLC may become even more important in the future, as novel and more effective systemic therapy (20–22) may be incorporated into the management of LA-NSCLC (23) and attenuate the competing risk of distant metastasis.

Radiotherapy dose escalation or intensification using altered fractionation has been shown to improve disease control in cancers of the prostate (24) and head and neck (25). Altered radiotherapy fractionation for LA-NSCLC has also been shown to improve outcomes to some extent in large, randomized clinical trials (26). Established radiobiological principles indicate that intensified radiotherapy is required to sterilize lung tumors, where hypoxia and accelerated repopulation contribute to radioresistance (27). For early stage lung cancer, hypofractionated stereotactic body radiotherapy (SBRT) yields excellent control rates, particularly when high biologically effective doses are delivered (28, 29). Advances in radiotherapy treatment planning and delivery should be leveraged in a similar fashion to safely deliver curative radiotherapy doses for LA-NSCLC. While RTOG 0617 demonstrated that radiotherapy dose escalation applied to large volumes using conventional fractionation does not improve outcomes in LA-NSCLC (6), more innovative strategies to intensify radiotherapy using adaptive planning (30), SBRT boost (31), and particle therapy (32, 33) must be explored to improve outcomes for patients with LA-NSCLC.

### Supporting ALARA

Distant metastasis occurs within two years in the majority of LA-NSCLC patients who are treated with concurrent chemoradiotherapy with definitive intent (6). Thoracic radiotherapy, which can cause profound acute (34, 35) and subacute (36, 37) toxicities in a dose-dependent fashion, should therefore be administered cautiously in this patient population. The current “standard” schedule of 60 Gy in 30 fractions was established approximately 40 years ago in a landmark randomized trial (38). 60 Gy was chosen over 50 Gy because 60 Gy yielded slightly better (but not statistically significantly superior) outcomes with respect to overall survival and local disease control. The relevance of these findings to current practice, where LA-NSCLC patients are treated with vastly more advanced techniques and typically receive concurrent chemotherapy, is unclear.

Several retrospective studies demonstrated strong associations between radiotherapy dose and overall survival duration, including in patients receiving concurrent chemotherapy (39, 40). In light of the results of RTOG 0617, however, it appears likely that those associations are attributable to selection biases (e.g., treating smaller volume disease with higher doses) or advances in treatment techniques (41) and systemic therapy (42) that took place during the era when non-randomized dose escalation trials were performed. Notably, RTOG 0617 (6) and several other trials where chemoradiotherapy was intensified using altered radiotherapy fractionation (9, 10) failed to demonstrate that local disease control or overall survival is improved with more aggressive radiotherapy. Meta-analyses strongly suggest that radiotherapy intensification may be beneficial when radiotherapy is delivered without chemotherapy but has not improved outcomes in the setting of concurrent chemotherapy (9, 10). The ability to control LA-NSCLC with chemoradiotherapy may more closely be related to tumor biology (43) and disease burden (44) than with radiotherapy dose. One may therefore argue that clinical trials should seek to define the lowest radiotherapy dose that can be used to treat LA-NSCLC without meaningfully compromising the likelihood of local disease control. An adaptive study design, such as the time-to-event continual reassessment model (45) could be could be ideal for defining a “minimum tolerated dose” in this setting. Based on analyzes of recurrence patterns demonstrating that local disease progression typically occurs in regions with large initial disease burden (46), a dose-painting approach may be implemented to reduce the dose delivered to small tumors and lymph nodes. In the rare cases where isolated thoracic disease progression occurs, salvage treatment options such as SBRT may yield excellent rates of disease control with acceptable toxicity rates (47).

## Fact: Serious Complication Rates Following Thoracic Radiotherapy for LA-NSCLC are Low

### Supporting MTD

The elimination of elective nodal irradiation (48), advances in imaging and target delineation (49), and advances in treatment techniques have significantly reduced the toxicity profile of thoracic irradiation (50). Two dose escalation studies demonstrated that treatment with 74 Gy in 37 daily fractions with concurrent carboplatin and paclitaxel is safe (51, 52), leading to the use of that regimen in the experimental arm of RTOG 0617. In RTOG 0617, rates of severe (grade ≥ 3) toxicities were essentially equal across the control (60 Gy) and experimental (74 Gy) arms, demonstrating that modern treatment techniques and evidence-based constraints can be implemented to allow the safe delivery of dose-escalated thoracic radiotherapy. Complication rates may be expected to decline in future trials, as intensity-modulated radiotherapy and particle radiotherapy are increasingly being implemented for the treatment of LA-NSCLC (32, 53). Esophagitis is one important acute complication of thoracic radiotherapy that occurs in a dose-dependent fashion (6, 34). With modern treatment techniques and supportive care measures, however, most patients can complete radiotherapy without a treatment break (54).

### Supporting ALARA

Evolving evidence reveals that thoracic irradiation can have profound consequences that were previously not appreciated. Two examples are provided below. As these risks emerge in a dose-dependent fashion, it is imperative that we examine the relationship between radiotherapy dosing and outcomes in LA-NSCLC rigorously and without bias and implement the lowest dose required to achieve local disease control.

Across the field of Oncology, patient-reported outcomes (PROs) have emerged as a key tool for assessing individual patients as well as in evaluating novel treatment strategies. PROs may be particularly revealing in the setting of LA-NSCLC, where patients’ health status may be compromised by underlying comorbidities, disease burden, and treatment toxicity. In a key secondary analysis of RTOG 0617, treatment with 74 Gy rather than 60 Gy dramatically increased the risk of meaningful quality of life decline at 3 months (55). Baseline quality of life scores were also found to be significant predictors of overall survival on multivariable analyses. The “safety” of high-dose thoracic radiotherapy should be reexamined using PROs. Existing data indicate that patient-reported toxicity rates will differ dramatically from clinician-scored adverse event rates (56), particularly in the setting of dose-escalated radiotherapy.

A growing body of literature indicates that minimizing cardiac irradiation should be a goal in planning thoracic radiotherapy. Recent publications have demonstrated a strong association between cardiac irradiation and both cardiac events (36, 37, 57, 58) and all-cause mortality (6). The risks of cardiac irradiation may be highest in subjects with comorbid conditions such as existing heart disease (36, 37) or a smoking history (58), which are common in NSCLC patients. Somewhat surprisingly, these effects have been seen within a few years of radiotherapy delivery (6, 36, 37, 57) and in populations with high risk of cancer-specific mortality (6, 36, 37). In retrospect, this is consistent with previous analyses demonstrating that excessive (59) or unnecessary (60) mediastinal irradiation for lung cancer can meaningfully reduce survival rates. Thoracic irradiation may directly lead to coronary artery stenosis (61) and may also impair patients’ immune systems (62).

## Fact: Systemic Therapy for NSCLC is Evolving Rapidly

### Supporting MTD

Targeted therapy and immunotherapy are revolutionizing the management of advanced NSCLC (20, 63). As these agents are incorporated into the management of LA-NSCLC (23, 64), one may expect the rate of distant metastasis to improve significantly. This could magnify the importance of achieving durable local disease control with effective radiotherapy. If induction therapy is utilized to reduce target volumes before delivery of thoracic radiotherapy, dose escalation with conventional or even stereotactic radiotherapy techniques would be particularly appealing.

Immunotherapy may be an ideal partner for high-dose radiotherapy. Radiotherapy may enhance tumor antigen presentation, increase cytokine production, and modulate the tumor microenvironment, promoting antitumor immunity (65, 66). Numerous preclinical studies (67, 68) and case reports (68–71) have demonstrated that there may be synergy between radiotherapy and immunotherapy. These effects may be maximized by employing “ablative” radiotherapy schedules, avoiding prolonged treatment courses, minimizing incidental irradiation of regional lymph nodes and other organs, and utilization of heavy ion radiotherapy (72, 73).

### Supporting ALARA

For appropriately selected patients with advanced NSCLC, targeted therapy (74) or immunotherapy (20) yields far higher response rates and more durable disease control than cytotoxic chemotherapy. If similar responses are seen in LA-NSCLC, relatively low radiotherapy doses may be required to provide high rates of local disease control. On a patient level, tumor characterization and molecular subtyping will be facilitated by liquid biopsies (75). Functional imaging (46, 76) and radiomic analyses (77, 78) will also aid in identifying patients and specific tumors or lymph nodes where disease is likely to be controlled without receiving high radiotherapy doses. It is imperative that radiation oncologists continuously reassess the relationship between radiotherapy dose and local disease control, as NSCLC is increasingly understood represent a mosaic of heterogeneous diseases rather than a single disorder. At the same time, novel systemic agents may unexpectedly modulate the toxicity profile of thoracic radiotherapy (79, 80) such that modest radiotherapy doses optimize the risk/benefit ratio of thoracic irradiation for LA-NSCLC. Therefore, the relationship between radiotherapy doses and toxicity risk must also be reassessed frequently, preferably in trials designed to account for subacute and delayed adverse events (81).

## Conclusion

Decades of clinical trials have not changed in the “standard” radiotherapy dosing for LA-NSCLC. However, it remains unlikely that current practices yield optimal results, and it is impossible to believe that a single dosing regimen should be administered to every patient with LA-NSCLC. The effects of radiotherapy dosing on both disease control probability and complication probability must be reassessed as new systemic treatment options emerge and as new subtypes of NSCLC are recognized. PROs may provide more meaningful information that physician-scored toxicity rates and should be incorporated into all NSCLC trials.

## Author Contributions

The lead author (NO) was responsible for designing and writing this review article.

## Conflict of Interest Statement

The author declares that the research was conducted in the absence of any commercial or financial relationships that could be construed as a potential conflict of interest.
